# Xenobiotics Delivered by Electronic Nicotine Delivery Systems: Potential Cellular and Molecular Mechanisms on the Pathogenesis of Chronic Kidney Disease

**DOI:** 10.3390/ijms231810293

**Published:** 2022-09-07

**Authors:** Pablo Scharf, Felipe Rizzetto, Luana Filippi Xavier, Sandra Helena Poliselli Farsky

**Affiliations:** Department of Clinical and Toxicological Analyses, School of Pharmaceutical Sciences, University of Sao Paulo, São Paulo 05508-220, Brazil

**Keywords:** diabetes mellitus, hypertension, nicotine, risk assessment, flavoring agents, volatiles organic compounds, cigarette smoking, ENDS

## Abstract

Chronic kidney disease (CKD) is characterized as sustained damage to the renal parenchyma, leading to impaired renal functions and gradually progressing to end-stage renal disease (ESRD). Diabetes mellitus (DM) and arterial hypertension (AH) are underlying diseases of CKD. Genetic background, lifestyle, and xenobiotic exposures can favor CKD onset and trigger its underlying diseases. Cigarette smoking (CS) is a known modified risk factor for CKD. Compounds from tobacco combustion act through multi-mediated mechanisms that impair renal function. Electronic nicotine delivery systems (ENDS) consumption, such as e-cigarettes and heated tobacco devices, is growing worldwide. ENDS release mainly nicotine, humectants, and flavorings, which generate several byproducts when heated, including volatile organic compounds and ultrafine particles. The toxicity assessment of these products is emerging in human and experimental studies, but data are yet incipient to achieve truthful conclusions about their safety. To build up the knowledge about the effect of currently employed ENDS on the pathogenesis of CKD, cellular and molecular mechanisms of ENDS xenobiotic on DM, AH, and kidney functions were reviewed. Unraveling the toxic mechanisms of action and endpoints of ENDS exposures will contribute to the risk assessment and implementation of proper health and regulatory interventions.

## 1. Introduction

Chronic kidney disease (CKD) is a silent outcome that occurs due to the aging process as a consequence of metabolic and vascular diseases. It occurs due to a sustained damage of renal parenchyma evolving to the chronic deterioration of renal function, which may gradually progress to end-stage renal disease (ESRD) [1]. CKD affects about 13.4% of inhabitants worldwide, leading to severe morbidity and mortality. It is more prevalent in people older than 65; nevertheless, the expectancy of progression to ESRD is higher when the disease manifests itself in younger people [2]. The aging of an organism proceeds at variable rates, being influenced by gene background, lifestyle, environmental exposure, and habits. Therefore, the etiology and incidence of CKD varies widely worldwide [3]. The disease is more prevalent in the low socioeconomic status population, and racial/ethnic/low-class minority groups in high-income countries [4]. CKD overcharges health systems worldwide, as all stages of the disease can be responsible for the impaired quality of life and the premature death of patients [5].

CKD presents a well-defined progression; however, prevention of ongoing disease is still a challenge, as symptoms occur during the latter phase of disease and early sensitive and specific biomarkers are not available. The progression of CKD, before reaching ESRD, can last for years [6]. Robust data associate CKD to previous type II Diabetes Mellitus (DM) and arterial hypertension (AH). Indeed, about 20% to 30% of DM patients evolve to ESRD, and hypertensive patients are around 15 times more likely to develop ESRD than normotensive individuals [7,8].

Long-term exposure to environmental pollutants may lead to silent toxic effects, where symptoms are manifested at the late progression of diseases [9,10]. Cigarette smoking (CS) is responsible for severe health problems and is pointed out as the major cause of preventable deaths worldwide. It is expected that cigarette smoking causes about 7.5 million deaths/year and burdens individuals and health systems, achieving up to US $500 billion globally per year, with costs of productivity loss, illnesses and premature deaths [11]. Although massive tobacco control policies have been successful, with marked reductions in the estimated prevalence of daily smoking in the last 20 years, the number of smokers increased significantly at the global level because of population growth. Beyond that, in some countries and territories, such as China, Albania, Portugal and Latin America, smoking prevalence among adolescents of both genders has augmented substantially and represented a steady increase among young people [11,12,13]. CS is a severe concern to CKD onset and progression and growing epidemiological studies have pointed out an increase incidence of CKD among the healthy population or diabetic subjects [14,15,16,17,18,19,20].

DM incidence, especially type II, has increased worldwide during the last decades, generating a tremendous impact on public health systems [21]. Exposure to environmental pollution is associated with DM prevalence [22], including CS [23,24]. Several epidemiological studies demonstrated the high incidence of pre-diabetes or DM in heavy smokers. Furthermore, quitting smoking reduces the estimative of occurrences of the disease, especially ten years after stopping tobacco addiction [4]. DM leads to damage of blood vessels, with consequent macro and microvascular complications, which are worsened by smoking [25]. Therefore, smoking is a pivotal modifiable risk factor to prevent the onset or delay the DM complications [26]. Moreover, several studies associate the risk of smoking during pregnancy or feeding on insulin resistance and DM in the offspring. Hypoxia, oxidative stress, and inflammation in the uterus microenvironment of smoker mothers lead to epigenetic alterations related to DM genesis [27].

AH is also a highly prevalent disease and is one of the leading causes of premature death worldwide [28]. Unquestionably repeated exposure to combustible product released by CS leads to AH and can display different mechanisms on AH genesis [29], such as (I) modifying the metabolism and the reactivity of endothelial and muscles of vessels wall [30]; (II) causing systemic inflammation and atherosclerosis; (III) oxidative stress [31]; (IV) impairing renal filtration which activates the renin-angiotensin and aldosterone system [32]; and (V) activating the sympathetic nervous system and hypothalamus-pituitary-adrenal gland axis with consequent release of vasoconstrictors [33].

Based on the incidence of DM and AH in smokers, one could expect a positive correlation between smoking and CKD. Indeed, smoking is an inducer and accelerating agent on the progression of established CKD in adults and young people, and smoking is considered a modifiable lifestyle factor for the prevention of CKD [34,35]. Moreover, products released by CS cause direct noxious effects in renal cells, and the main mechanisms are an unbalanced redox system and induction of inflammation. The intracellular downstream effects elicited by reactive oxidative species (ROS) and inflammatory mediators amplify the inflammatory process and activate cellular death pathways in renal cells [36,37].

The toxicity of CS is associated with the release of nicotine and products generated during the combustion process, which reached temperatures around 650 °C. It generates more than 8000 compounds, and almost 70 are classified as carcinogens [38]. Indeed, tobacco-released products can trigger several deleterious effects and impair multiple function, such as hematopoiesis [39], autoimmune and inflammatory responses [40,41], metabolism [42], respiratory functions [43] and so on. As a possible harm reduction tool to current smokers, tobacco products lacking the combustion process have emerged worldwide [44,45]. These products named electronic nicotine delivery systems (ENDS) include e-cigarettes, pods, and heated tobacco products that can release nicotine and other chemicals by a heating process [46]. However, knowledge about the toxicity of ENDS is still limited, and further experimental and epidemiological data are required for the risk assessment of the possible chronic effects of these devices and their possible association with diseases onset, such as CKD. Although ENDS lack combustion, they can release nicotine, byproducts from thermal degradation (acrolein and carbonyl compounds), and flavoring agents that can trigger potential toxic effects related to several pathological conditions [45,47].

Based on the growing consumption of tobacco products, including ENDS, here we review the current knowledge of the mechanism underlying the toxic actions of xenobiotics released by ENDS and their role on CKD pathophysiology. We focused on the role of nicotine, solvents employed as vehicles, and toxic substances generated by ENDS devices in the genesis of CKD, centering the attention on mechanisms of DM, AH, and the damage of renal cells.

## 2. Electronic Nicotine Devices Systems (ENDS): A Rising Concern for CKD Pathogenesis?

Based on the recognized hazardous effects of tobacco combustion, a wide array of nicotine replacement therapies has been used to aid tobacco cessation in a therapeutic context for over 30 years, including gums, transdermal patches, nasal spray, oral inhalers, and tablets [44]. The first ENDS emerged in the 60′s decade also as an alternative to delivering nicotine by airways, in pulses, as occurs in cigarette addiction. They were proposed as harm reduction agents; nevertheless, the enormous technological advances in vaping nicotine and a increased number of commercialized devices led to widespread employment over the world in the last decade [46].

All ENDS devices have a rechargeable battery, and, in some cases, an atomizer is necessary to heat the nicotine content. Nicotine can be stored in a cartridge filled with a liquid that can be exchanged, or in special tobacco sticks, known as heat-not-burn tobacco systems [45]. Both systems use glycerin and propylene glycol as humectants; nevertheless, several flavorings can be used for commercial appeal. In some cases, liquids and cartridges are filled with flavorings without nicotine [47].

Conventional cigarettes burn tobacco and generate smoke and ashes during the combustion process; differently, ENDS are heated at much lower temperatures when compared to conventional cigarettes, generating vapor [48]. Although ENDS deliver much lower amounts of tobacco combustion products, the heating process can generate byproducts from thermal degradation or compounds released from batteries supply, for which toxicity has not been described [45]. Indeed, recent epidemiological evidence has provided controversial data, such as on AH [49,50] as DM [51,52].

Considering the importance of CKD as a public health problem and the growing employment of ENDS worldwide, it is pivotal to evaluate if such exposures can evoke cellular and molecular mechanisms related to CKD onset and progression. Notably, further experimental, clinical, and epidemiological data about the acute and chronic use of ENDS are required to assess their real toxicity. Therefore, we selected the main compounds delivered by ENDS devices and depicted their potential toxic mechanisms involved in CKD pathogenesis.

## 3. Nicotine

Nicotine (3-(1-methyl-2-pyrrolidinyl) pyridine) is a water-soluble alkaloid and the main component found in tobacco leaves [53]. Nicotine is promptly absorbed by the lungs and biodistributed. Pharmacokinetics studies designed to assess the nicotine uptake by electronic aerosol devices showed similarities in nicotine absorbed from conventional cigarettes. Both caused a rapid increase in plasma concentration (minutes) with similar Tmax and Cmax, and a half-life of around 60–90 min [54]. About 80% of nicotine is metabolized to cotinine in the liver microsomes, mainly by the isoform CYP2A6 enzyme; cotinine is further metabolized by the same enzyme to trans-3-hydroxycotinine. Furthermore, nicotine is also metabolized to norcotinine via N-demethylation by CYP2A6 and CYP2B6 at low and high substrate concentrations, respectively. Gene polymorphism of CYP2A6 leads to differences in nicotine metabolism and smoking behavior [55].

Nicotine binds to nicotine acetylcholine receptors (nAChRs), widely distributed in central and peripheral nervous systems and on a diversity of peripheral cells, such as including epithelium, endothelium, immune (neutrophils, monocytes, macrophages, DC, B cells, and T cells), cancer, astrocytes, and oligodendrocytes [56,57,58,59]. nAChRs are transmembrane pentameric ligand-gated ion channel members of the Cys-loop superfamily. The receptor comprises five distinct subunits, which form a central aqueous pore that allows cation (Ca^2+^, Na^+^, K^+^) transportation, an extracellular domain that binds to ligands, a transmembrane and an intracellular domain [60,61]. Agonist binding on nAchR displays rapid conformational changes on the three-dimensional structures of the receptors. It leads to ion channels opening to the influx of cations and rapid cell depolarization [60]. Seventeen subunits of nAChRs are characterized, in which 12 subunits are known neuronal-type nAChRs (α2–α10 and β2–β4) and five subunits are muscle-type nAChRs (α1, β1, δ, γ and ε) [62]. Beyond their channeling activity, nAChRs activation mediates diverse intracellular events involving signaling through PI3-kinase, ERK1/2, AKT, and CREB pathway, and mediates proteolysis and mitochondrial permeability [63]. The extraordinary diversity and distribution of nAChR isoforms, associated with different affinities to ligands, results in numerous functional responses to agonists [57,58,59,64]. In physiological conditions, nAChRs bind to acetylcholine and trigger neuron depolarization and the parasympathetic autonomic nervous system in the peripheral systems [65]. It displays pivotal roles in the homeostasis of different systems, such as cardiovascular, respiratory, central nervous, and immune [66]. In comparison to acetylcholine, nicotine causes longstanding activation of nAchR, accompanied by rapid receptor desensitization [60,67]. Therefore, chronic nicotine exposure causes adaptations on the central nervous system, including the upregulation of nAChR subtypes. As a consequence, a withdrawal syndrome develops during attempts to quit smoking.

Data have associated nicotine exposure and AH incidence and pointed out different and interconnected mechanisms due to the high distribution and diversity of nAchR on cells involved in the complex genesis of AH, such as:

(I) Different mechanisms of action of nicotine on the vascular and cardiac autonomic nervous system have been broadly shown. For instance, nicotine directly affects the cardiac autonomic nervous system and mediates cardiac autonomic ganglion transmission [68,69]. Notably, nicotine during pregnancy results in autonomic dysfunction and increased blood pressure in the offspring, leading to structural–functional modifications in the arterial wall and heart [70]. Nicotine also impairs the baroreflex sensitivity to sodium nitroprusside by disrupting adenosine A(2A) receptor-mediated facilitation of reflex cardiac sympathetic excitation [71];
(II) Activation of nAChRs in adrenal gland chromaffin cells induces the release of catecholamine into blood, which acts in peripheral vessels receptors and elevates the blood pressure [72];
(III) Long-lasting exposure to nicotine affects target genes correlated to blood pressure control. Elevation of the blood pressure in mice by nicotine administration was associated with reduced expression and activity of renal 11β-hydroxysteroid dehydrogenase type 2 (11β-HSD2) in renal cortical collecting duct cells. 11β-HSD2 catalyzes the conversion from active into inactive glucocorticoids and favors the expression and occupancy of mineralocorticoids receptors. The suppression of 11β-HSD2 by nicotine was dependent on the suppression of C/EBPβ (CCAAT/enhancer-binding protein-β) and activation of Akt protein kinase phosphorylation (pThr308Akt/PKB) by kidney cells [73]. Furthermore, exposure to oral nicotine for 28 days increased blood pressure, impaired glomerular filtration rate and fraction excretion of sodium, and augmented sympathetic cardiac modulation in mice with reduced renal Klotho gene expression [74]. The gene encodes a single-pass transmembrane protein expressed predominantly by the distal convoluted tubules in the kidneys and is related to calcium-phosphorus metabolism, ion channel regulation, intracellular signaling pathways, and longevity. Klotho deficiency is associated with a syndrome that resembles human aging, acute kidney injury and renal fibrosis. Moreover, the overexpression or replacement of klotho protects against or ameliorates such injury [75,76]. Therefore, the association of nicotine actions on the expression of the Klotho gene by distal tubules cells deserves further investigation to provide knowledge to the comprehension of nicotine on the genesis of AH and CKD;
(IV) Nicotine affects the mechanisms of endothelial cells on the control of blood pressure and leads to kidney inflammation. Chronic exposure to nicotine by an oral route further increased the blood pressure of rats evoked by high-diet obesity, related to endothelial activation and inflammation. In this context, nicotine augmented the expression of superoxide in endothelial cells and impaired endothelial nitric oxide synthase (eNOS), an endothelial-derived relaxing factor. Indeed, several nAChR subunits are identified in endothelial cells, and their activation leads to downstream signaling that mediates endothelial proliferation, survival, migration, angiogenesis, blood pressure, and inflammation [77,78]. Furthermore, peritoneal macrophages of nicotine-treated obese rats further released tumor necrosis factor (TNF) α and interleukin (IL) 1β and presented higher expression of CD36 [79]. In addition, the role of systemic nicotine as an inducer of renal inflammation was shown, as a continuous infusion of nicotine for two weeks in spontaneously hypertensive rats caused marked infiltration of CD161a^+^ monocyte into kidneys and premature hypertension. The renal infiltration of cells was correlated to the augmented secretion of the chemotactic cytokine monocyte-chemoattractant-protein-1 (MCP-1) and the membrane expression of the adhesion molecules very-late antigen-4 (VLA-4) and CD161a ligand Lectin-LikeTranscript-1 (LLT1) by renal cells [32];
(V) Nicotine alters the renin-angiotensin-aldosterone system (RAAS) homeostasis. Chronic nicotine administration elevated plasma renin activity in rats subjected to a high-salt diet, and nicotine administration increased plasma angiotensin-converting enzyme activity, with consequent enhancement of conversion of angiotensin I to angiotensin II [80]. Moreover, nicotine exposure inhibited aldosterone production by the adrenal gland, which led to compensatory RAAS activation upon chronic exposures [81,82]; and
(VI) Nicotine modulates the cholinergic central nervous system and activates the hypothalamic-pituitary-adrenal (HPA) axis, which increases blood pressure by sympathetic stimulation [83].

The fine-tuning mediation of nicotine on neuroendocrine systems is extensively shown. In this way, nicotine displays several mechanisms on glucose and insulin homeostasis and may cause dual opposite effects dependent on concentrations and lasting exposures [84].

As previously described, nAchR activation induces catecholamines release and HPA activation [72,85] Secreted catecholamines mobilize glucose primarily from stored skeletal muscle and liver glycogen [85]. Augmented levels of cortisol in the blood cause: (I) liver gluconeogenesis, with the consequent decrease on glucose uptake; (II) a permissive role for catecholamine-induced glycogenolysis and/or inhibition on insulin-stimulated glycogen synthesis; (III) modulation on functions of pancreatic α and β cells to regulate the secretion of glucagon and insulin. Therefore, elevated catecholamines and glucocorticoids levels lead to hyperglycemia and insulin resistance [85].

More recently, it has proposed a novel pathway on glucose control by nicotine in the brain, by the interconnection of diabetes-associated transcription factor 7 like 2 (TCF7L2) and nAchRs in the ventral region of the medial habenula. Indeed, the medial habenula is a major cholinergic pathway in the brain [86] that maintains a polysynaptic connection with the pancreas [87], and expresses TCF7L2. Activation of medial habenula by nicotine disrupts blood glucose homeostasis, reflected by elevated fasting blood glucose and glucagon levels, which was not detected in animals deficient in TCF7L2 [88].

Islet β cell senescence is a hallmark of DM. Senescent β cells permanently loose proliferative capacity. They are characterized by shortened telomeres, higher expression of senescent markers, such as p16, p21, and p19, elevated secretion of multiple pro-inflammatory cytokines, including TNF, IL-6, MCP1, and IL-1β, increased cell size, high glucose uptake, and mitochondrial dysfunction [89]. Direct nicotine exposure led to β cells senescence by local accumulation of reactive oxygen species (ROS) [90], reinforcing the ability of nicotine to induce oxidative stress, as yet had been proposed as a toxic mechanism in other tissues [91,92,93]. Nevertheless, the hazardous role of nicotine on Islet β cells may depend on concentration and schedule of exposures. Stimulation of nAchR by nicotine, especially α7nAChR, increased insulin secretion and reduced cytokine-induced apoptosis in human and murine islet cells [94]. Moreover, in vivo administration of nicotine ameliorated the diabetic phenotype in rodent obese models and type I DM [95,96]. A proposed mechanism to these beneficial effects is the re-establishment of cell homeostasis, by modulating the stress endoplasmic reticulum and activation of inositol requiring enzyme 1α [97].

Nicotine is the tobacco compound most highly correlated with metabolic dysfunction in offspring of tobacco addict mothers. Exposure of pregnant mice to nicotine caused several systemic effects in the mother and offspring, including insulin resistance [98,99,100]. Robust investigations support that nicotine intake leads to glucocorticoid overexposure in the mother. Exacerbated glucocorticoid actions lead to intrauterine neuroendocrine programming changes in offspring switching them susceptible to metabolic diseases [98,101]. Moreover, chronic nicotine exposure to dams causes oxidative stress and toxicity in target cells. It promotes post-translational histones in perivascular adipose tissue and β-cells [102,103]. Recently, it was found that a pre-diabetic state in offspring of nicotine-exposed dams was associated with downregulated transcription factor sterol regulatory element-binding protein-1c (SREBP-1c), peroxisome proliferator-activated receptor-α (PPAR-α), and insulin receptor in the liver [99,104]. SREBP-1c transduces the insulin signal and induces the expression of a family of genes involved in glucose utilization, fatty acid synthesis, and PPAR-α regulates glucose synthesis during fasting states and gluconeogenesis [105].

Beyond evoking mechanisms related to DM or AH, nicotine directly acts on kidney cells. Indeed, nAchR isotypes are highly distributed in the kidney [106,107]. Some investigations show α7nAChR activation by agonists protects renal against ischemia/reperfusion injury by eliciting anti-inflammatory actions [106,107,108]. Conversely, in vitro and in vivo investigations describe the toxicity of nicotine in renal cells, mainly by triggering oxidative stress and inflammation. These effects impair the viability and function of renal tubular and endothelial cells, alter renal hemodynamics, and compromise overall kidney function [109,110].

The connection between immune cells and cholinergic innervation in kidney lesions was demonstrated in different experimental models of spontaneously hypertensive rats. Administration of nicotine-induced premature hypertension, renal expression of the sodium-potassium chloride co-transporter, increased renal sodium retention, and an influx of CD161a^+^/CD68^+^ macrophages into the renal medulla. Bilateral renal denervation and depletion of CD161a^+^ immune cells abolished the toxicity caused by nicotine [32,111]. Moreover, sustained elevated expression of the unphosphorylated form of signal transducer and activator of transcription-3 (U-STAT3) was detected in kidneys of nicotine-exposed mice and was related to the sustained transcription of genes linked to remodeling and inflammation in the kidney during injury [112,113]; and incubation of nicotine with human cell kidney led to ROS generation and activation of NLRP6 inflammasome and endoplasmic reticulum (ER) stress [114].

Nicotine exposure exacerbates diabetic nephropathy, which is a leading cause of CKD. Expansion of mesangial, a precursor of glomerular sclerosis, is a hallmark of diabetic nephropathy [115]. Also, nicotine exposure further enhances the mesangial cell proliferation caused by hyperglycemia, dependent on enhanced Wnt/β-catenin downstream in primary human renal mesangial cells [116]. Indeed, DM leads to up activation of Wnt1/β-catenin signaling. The activation of the signaling pathway promoted podocyte injury, the epithelial–mesenchymal transition of podocytes, along with renal injury and fibrosis [117,118]. Moreover, nicotine binds to podocytes, causes an unbalance of the redox system, and activates inflammatory and apoptotic pathways. DM mice exposed to nicotine presented proteinuria and reduced glomerular podocyte synaptopodin. Synaptopodin is a crucial stabilizer of the podocyte cytoskeleton [119]. As podocytes are pivotal to maintaining the structure and function of the glomerular filtration barrier, the hazardous mechanisms described by nicotine actions assuredly contribute to CKD.

Considering nicotine released by CS and ENDS presents high bioavailability, prompt reaching peripheral and central systems, the mechanisms elicited above can be triggered by both tobacco products. It is pivotal novel epidemiological and experimental data that could clarify the real role of these novel tobacco devices on the genesis of AH, DM, and CKD. Indeed, e-cigarette consumption caused alteration in the renal functions with changes in pivotal parameters, such as urea and creatinine, histological changes in the renal tissue. Altogether, these symptoms point out the possible nephrotoxic effects due to nicotine delivered by these devices [120].

## 4. ENDS Vehicles and Its Thermal Degradation Byproducts

Humectants are employed in ENDS devices to generate aerosols/vapor upon the heating process. Propylene glycol (PG) and vegetable glycerin (VG) is the most common vaporizing carriers employed in these devices. They favor the delivery of flavorings and nicotine [121]. The addition of PG and VG is widely employed in the food industry, presenting almost no toxicity when used for ingestion, however, when there are modifications in the route of administration, toxic effects are observed. In vitro studies show PG or VG alters cell growth and survival and impairs DNA repair functions [122,123]. On the other hand, intramuscular injection of glycerol is used as an experimental model of rhabdomyolysis-induced acute kidney injury, which is reduced by anti-oxidant administration [124]. Upon the heating process, PG and VG can generate byproducts with recognized toxicities, including reactive aldehydes and volatile organic compounds, such as acrolein, acetaldehyde, and formaldehyde [125]. It is noteworthy to mention, these later compounds are also found in the CS.

Acrolein is a highly reactive unsaturated aldehyde, which displays dose-dependent toxicity. Acrolein is generated from the thermal degradation and reaction of glycerol, and its formation can increase by 28-fold when glycerol e-liquid composition reaches 80%, which is a current concern during ENDS exposures [126,127]. Acrolein exposures activate/inactivate several pathways related to AH, as follows: (I) acrolein activates the transient receptor potential ankyrin 1 (TRPA1), a non-selective cation channel, and increases vascular permeability and leukocyte extravasation, leading to microvascular endothelial dysfunction [128,129]; moreover, TRPA1 activation can also increase the release of the hypertensive neuropeptide Substance P [130]; (II) acrolein reacts with structural molecules and disturbs the cellular redox balance, not only by increasing ROS production but also by depleting the antioxidant enzyme glutathione [131]; (III) higher and chronic acrolein exposures can also suppress eNOS phosphorylation and induce the Activating Transcription Factor-2 (ATF-2) expression, which leads to increased expression of the angiotensin 1 receptor in arteries [132,133]. Indeed, experimental models exposed to acrolein displayed increased diastolic and systolic values, followed by higher blood pressure and arrhythmias [134].

Acrolein exposures and detection of its urinary metabolites N-acetyl-S-(3-hydroxypropyl)-L-cysteine (3-HPMA) and N-acetyl-S-(carboxyethyl)-L-cysteine (CEMA) present a positive correlation with the development of DM and insulin resistance [135,136]. Although the precise mechanism involved in the diabetogenic potential of acrolein remains unclear, these events are closely related to the capacity of acrolein to activate oxidative stress and systemic inflammation, which directly affects glucose metabolism and insulin resistance [133,136].

Acetaldehyde and formaldehyde are volatile compounds routinely related to carcinogenic outcomes [137]. However, both chemicals evoke hemodynamic alterations in experimental models, such as altered heart rate, increased blood pressure, and impaired cardiac contractibility [138,139]. Furthermore, these chemicals lead to oxidative burst, which triggers inflammatory responses and systemic toxic effects. Higher levels of acetaldehyde and formaldehyde are found in the course of metabolic syndromes and DM [140]. Although both compounds are generated under homeostasis, it was observed that the administration of exogenous formaldehyde was able to induce hyperglycemia in rats, followed by a deficiency in insulin signaling [139]. Formaldehyde exposures into experimental animals induced proximal tubule necrosis and increased the concentration of malonaldehyde, indicating the activation of redox signaling [141]. The latter observation was corroborated by an impaired renal function of formaldehyde-exposed rats, characterized by increased urea and creatinine levels, and altered renal structure caused by a marked enhancement in Bowman’s space [142].

## 5. Flavoring Agents

Flavorings are chemical substances constantly added to ENDS, surpassing more than 7000 flavors that are used to make products more attractive to consumers [143]. Although many of the added flavorings in ENDS are recognized as safe (GRAS) by the FDA, these compounds are safe for ingestion, but not for inhalation after heating. The heating processes to inhalation generates ultrafine and toxic particles from flavoring compounds, wherein absorption by the lungs directly leads to harmful systemic effects [144]. The most common flavors added to stimulate consumption reach a vast range of options, such as minty, sweet, fruity, peppery, and buttery flavors [145]. Other flavors are employed to enhance the user experience, which includes menthol, cinnamaldehyde, vanillin and diacetyl. Tobacco products containing menthol can reduce the irritation caused by nicotine and other aerosolized products, once menthol modulates sensorial perception, leading to deeper inhalation and exposure to higher amounts of nicotine and other toxic particles [146].

Menthol and cinnamaldehyde are flavoring agents shared with conventional and combustible cigarettes. Although the systemic effects of inhaled flavorings are still unclear, the presence of menthol and cinnamaldehyde in nicotine-containing devices directly affects nicotine metabolism, nAChR expression, and distribution [146,147,148]. Both cinnamaldehyde and menthol can inhibit the main nicotine-metabolizing enzyme, CYP2A6. In fact, impaired nicotine metabolism and clearance favor addictive behaviors, leading to increased tobacco consumption and exposure to the aforementioned xenobiotics [149,150].

Beyond the direct effects on nicotine metabolism, cinnamaldehyde can directly activate TRPA1, which is widely expressed in neural and non-neural cells, such as endothelial, myocytes, and renal tubular cells [151]. Clinical and translational reports show a deleterious effect led by TRPA1 activation [151,152]. TRPA1 expression is directly associated with more severe cases of renal tubular, the expression of the DNA damage and oxidative stress marker 8-hydroxydeoxyguanosine (8-OHdG), indicating that TRPA1 activation plays a pivotal role in renal dysfunction [151]. It was observed that TRPA1 activation evoked higher ROS production and activation of mitogen-activated protein kinase (MAPK) and nuclear factor *kappa* B (NFκB) pathways, which resulted in IL-8 secretion by the human proximal tubular cells HK-2 [152]; beyond the lung microenvironment, cinnamaldehyde present in e-cigarettes displays higher cardiotoxicity; in vitro experiments testing the cytotoxicity of cinnamaldehyde in endothelial and in human-induced pluripotent stem cell-derived cardiac myocytes (hiPSC-CMs) indicated cinnamaldehyde increases cell death and impairs cellular metabolism and functions [153,154]; cinnamaldehyde drives to increased DNA damage even in non-cytotoxic concentrations, which highlights its cumulative cell damage [155].

Diacetyl is a member of organic diketones and is widely employed in the food industry to add buttery-based flavors to food [156]. Diacetyl has been detected in several ENDS; in some e-cigarettes, the aerosols can reach a concentration of 239 μg/e-cigarette [157]. With the exponential growth of novel ENDS flavors, it was observed that out of 51 e-cigarettes tested, 47 had detectable concentrations of diacetyl or its substitutes [157]. Diacetyl is the most common example of safe flavoring by oral ingestion, but not for inhalation [156]. Although the effects of inhaled diacetyl on ENDS users remain elusive, retrospective studies assessing the occupational exposures to heated and inhaled diacetyl indicate its potential toxicity, especially to the airways [158,159]. Diacetyl can interact with structural proteins, especially arginine-rich proteins, causing cellular damage and leading to aggregated and misfolded proteins, which in turn can trigger autophagy-mediated cell death [160,161]. Diacetyl exposure also evokes higher ROS production, formation of the DNA adducts deoxyguanosine, and in some cases, causing cell death by excessive ROS-induced damage [160,162].

## 6. Ultrafine Particles

In addition to the toxic effects directly caused to the ENDS user, aerosolized substances can release toxic compounds into the environment and generate secondhand exposures to bystanders [163]. Although particulate matter (PM) from combustible cigarettes is widely discussed, in particular PM_2.5_, the ultrafine particles (≤0.1 µm in diameter) have emerged as a great public health problem regarding indoor air quality along with the increasing consumption of ENDS [164,165].

The knowledge of the effects of ENDS consumption on indoor air quality and the assessment of delivery particles to the users requires further investigation. Nevertheless, ultrafine particles emitted during ENDS use can increase up to 10-fold in comparison to basal levels, reaching 7.2 × 10^3^ to 6.2 × 10^4^ particles/cm^3^ [166]. The ultrafine particle emission by ENDS is affected by several factors, such as the ENDS device generation, heating temperature, device voltage, and also e-liquid/tobacco-based stick composition [167]. Higher voltage devices generate more particles than low voltage devices [168,169]. Besides, the chemical composition of ENDS directly influences the concentration of emitted particles, including nicotine content, flavors, and the PG/VG ratios, which evoke higher particle releases by increased concentrations of chemicals [166].

The toxicity of ultrafine particles is based on their size and charge. Most inhaled ultrafine particles can reach deeper regions of the lung than larger particles (1–2.5 µm) [170]. Besides, positively charged ultrafine particles are up to 40 times more likely to penetrate cells than negatively charged particles [171]. Ultrafine particles translocate through the alveolar epithelium through diffusion [171]. Once these particles reach the lung vasculature, or lymphatic circulation, the inhaled ultrafine particles can trigger systemic inflammation, leading to vascular dysfunction and accumulation in the liver and kidney to exert their toxic effects [170,172,173].

Ultrafine particles generated by ENDS and released in secondhand exposures can increase intracellular calcium in the vasculature, evoking contractile dysfunction and disturbing heart rate, leading to arrhythmias [173,174]. These deleterious effects are related to the onset of oxidative stress and the activation of inflammatory pathways. Indeed, the ROS production evoked by ultrafine particles reacts with NO and shortens its endothelial bioavailability [175,176,177]. Several studies reported that ultrafine particles increase blood pressure and lead to coagulation-related changes [176,177]. The direct interaction of ultrafine particles with endothelium also causes vascular inflammation and sustains atherosclerosis establishment [170].

Although the effects of ultrafine particles released by ENDS on carbohydrate metabolism and pathophysiological mechanisms of DM are still limited, studies have shown the toxic potential of ultrafine particles present in air on DM outcomes [178,179]. DM patients who inhaled ultrafine carbon-based particles showed changes in coagulation parameters, in addition to higher levels of platelet activation and IL-6, enhancing hemodynamic dysfunction [180,181]. Indeed, ultrafine particles present in air pollution increase the risk of AH and DM [176,181].

Based on the mechanism involved on the toxicity of ultrafine particles and how these pollutants can trigger CKD-based diseases, it is possible to consider that, once ENDS are a growing source of first and secondhand ultrafine particles, the cumulative exposure can favor CKD onset and also exacerbate cardiovascular and metabolic dysfunction.

## 7. Conclusions and Future Perspectives

**Figure 1:**
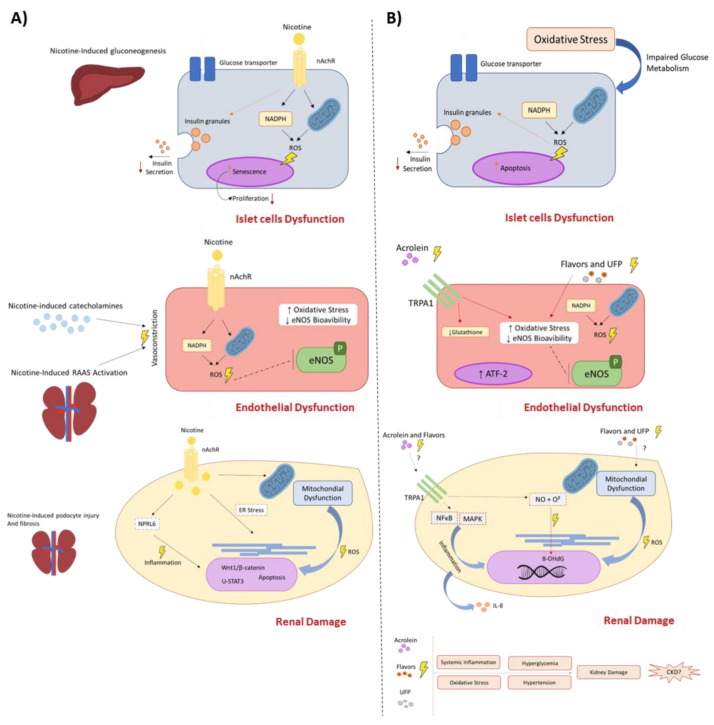
Potential cellular and molecular mechanism triggered by nicotine and compounds generated by ENDS and their role on CKD pathophysiology. ENDS release several chemical compounds, including nicotine, ultrafine particles, flavoring, and other harmful and potentially harmful constituents generated by thermal degradation. Thus, these compounds are absorbed by the lungs and reach systemic circulation to exert their toxicity and favor the onset of CKD and its related disorders. Although the harmful and direct effects of nicotine are well known and reported in clinical and experimental studies (**A**), other xenobiotics released by ENDS, such as acrolein, ultrafine particles (UFP), and flavorings possess the potential to trigger deleterious effects, leading to cumulative damages and favor CKD onset (**B**), however further data are required. The direct interaction of tobacco-released compounds with the endothelium increase reactive oxygen species (ROS), decreases endothelial nitric oxide species (eNOS) [73,74,126,127,128,129,170] and leads to an inflammatory response mediated or not by nicotinic receptor activation (nAChRs) or stress sensors receptors (TRPA1) [75,123,124,125,146,147], evoking endothelial dysfunction, impaired vascular relaxation, and increasing blood pressure. Directly or indirectly, these xenobiotics can impair glucose metabolism by affecting β islet cell homeostasis [85,86,90] and mitochondrial function in an ROS-dependent manner, culminating in insulin resistance and Diabetes Mellitus type II onset [95,100,135]. Finally, the kidneys are affected by the systemic inflammatory response, increased blood pressure, and higher levels of ROS, which increase mesangial cell proliferation, activate programmed cell death pathways on tubular cells, and impair mitochondrial function [109,110,111,112,113,114,115,135,136,137]. Once the kidneys are responsible for filtration, these xenobiotics accumulate on renal structures and cause inflammation and oxidative stress. Altogether, these mechanisms decrease glomerular filtration rate (GFR) and kidney function, leading to the late establishment of CKD.

The consumption of ENDS worldwide has grown exponentially, but the toxicity generated by chronic exposures to these devices is still unknown. Potentially harmful compounds released by ENDS can directly modulate cellular and molecular mechanisms related to chronic and multi-mediated disease onset, such as CKD. Although the precise mechanisms related to the interaction between ENDS use and CKD onset require further investigation, the possible pathways involved, and the xenobiotics released by ENDS are summarized in Figure 1. The elucidation of how ENDS and its released toxic substances can trigger systemic conditions which favor CKD onset and progression will provide novel data regarding the toxicity of non-combustible tobacco products, making it possible to improve the risk assessment policies and alert the investigations of CKD incidence in consumers.

## Data Availability

The data are contained within the article.
